# Supplementary material for: Open access resource for cellular-resolution analyses of corticocortical connectivity in the marmoset monkey
Source: Nat Commun. 2020 Feb 28;11:1133. doi: 10.1038/s41467-020-14858-0 (PMC7048793; doi:10.1038/s41467-020-14858-0)
Supplement: Supplementary file 1 — Supplementary Information [file 41467_2020_14858_MOESM1_ESM.pdf]

Open Access Resource for Cellular-Resolution Analyses of Corticocortical Connectivity in the Marmoset Monkey

Piotr Majka, Shi Bai, Sophia Bakola, Sylwia Bednarek, Jonathan M. Chan, Natalia Jermakow, Lauretta Passarelli, David H. Reser, Panagiota Theodoni, Katrina H. Worthy, Xiao-Jing Wang, Daniel K.Wójcik, Partha P. Mitra, Marcello G.P. Rosa

Supplementary Information

Contents:

Supplementary Figure 1: Sample characteristics with respect to the six different tracers used.
Supplementary Figure 2: Relationship between the number of labeled neurons and injection site volume.
Supplementary Figure 3: Calculation of geodesic paths and interareal distances.
Supplementary Figure 4: Comparison of connectivity distance analysis using different definitions of primary areas.
Supplementary Figure 5: Overview of the computational pipeline for mapping the results of the injections into stereotaxic coordinates and for obtaining quantitative connectivity patterns illustrated using an example case.
Supplementary Figure 6: Summary of the label maps delineated in both experimental cases as well as in the template, to increase the coregistration accuracy in the expert-assisted reconstruction step.
Supplementary Figure 7: The architecture of the http://www.marmosetbrain.org portal.

Supplementary Table 1: Abbreviations of the names of cortical areas.
Supplementary Table 2: Key materials and their sources.
Supplementary Table 3: Full list of injections.

Supplementary References

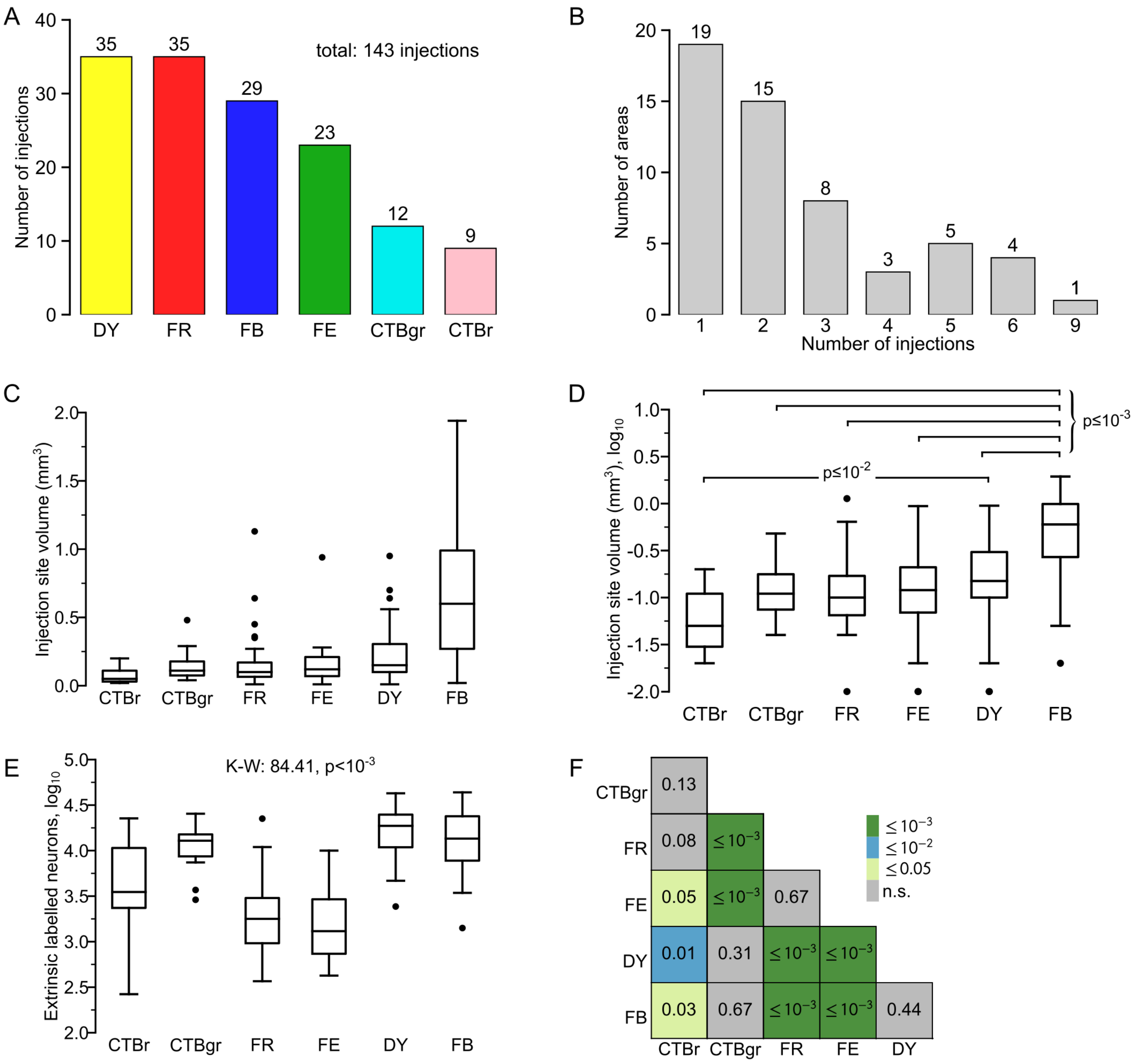

Supplementary Figure 1. Sample characteristics with respect to the six different tracers used (related to *Methods*, Surgical procedures). **A**: Numbers of successful injections using the different tracers included in the present sample. Injections with extensive contamination of white matter, or which did not result in long-range transport have been excluded from the sample. **B**: Number of injections centered in different areas. Most areas have received only one or two injections (19 and 15 areas, respectively). Other areas, such as V2 (9 injections), V1 and area 10 (6 injections each) have been injected multiple times. **C**, **D**: Distribution of injection volumes, in mm^3, shown in linear scale (**C**) and log10-transformed to mitigate the heteroskedasticity (**D**). The volume of the injection site depends on the tracer type (Kruskal-Wallis H test: 38.21, $p<10^{-3}$). Horizontal bars indicate statistically significant differences according to post-hoc Dunn's test. FB resulted in the largest injection sites, determined histologically[1], and CTBr the smallest. **E**: Number of neurons forming extrinsic connections, according to the type of tracer (Kruskal-Wallis H test: 84.41, $p<10^{-3}$). **F:** Pairwise differences between the number of neurons labeled by injection performed with different tracers according to post-hoc Dunn's test. FB and DY tracers tended to label a large number of neurons, together with CTBgr while FR and FE injections result in the smallest number. Box-plots on panels (**C**-**E**) show order statistics (center line: median; box limits: upper and lower quartiles; whiskers: $1.5\times$interquartile range; points: outliers) of the corresponding values. Source data are provided as a Source Data file.

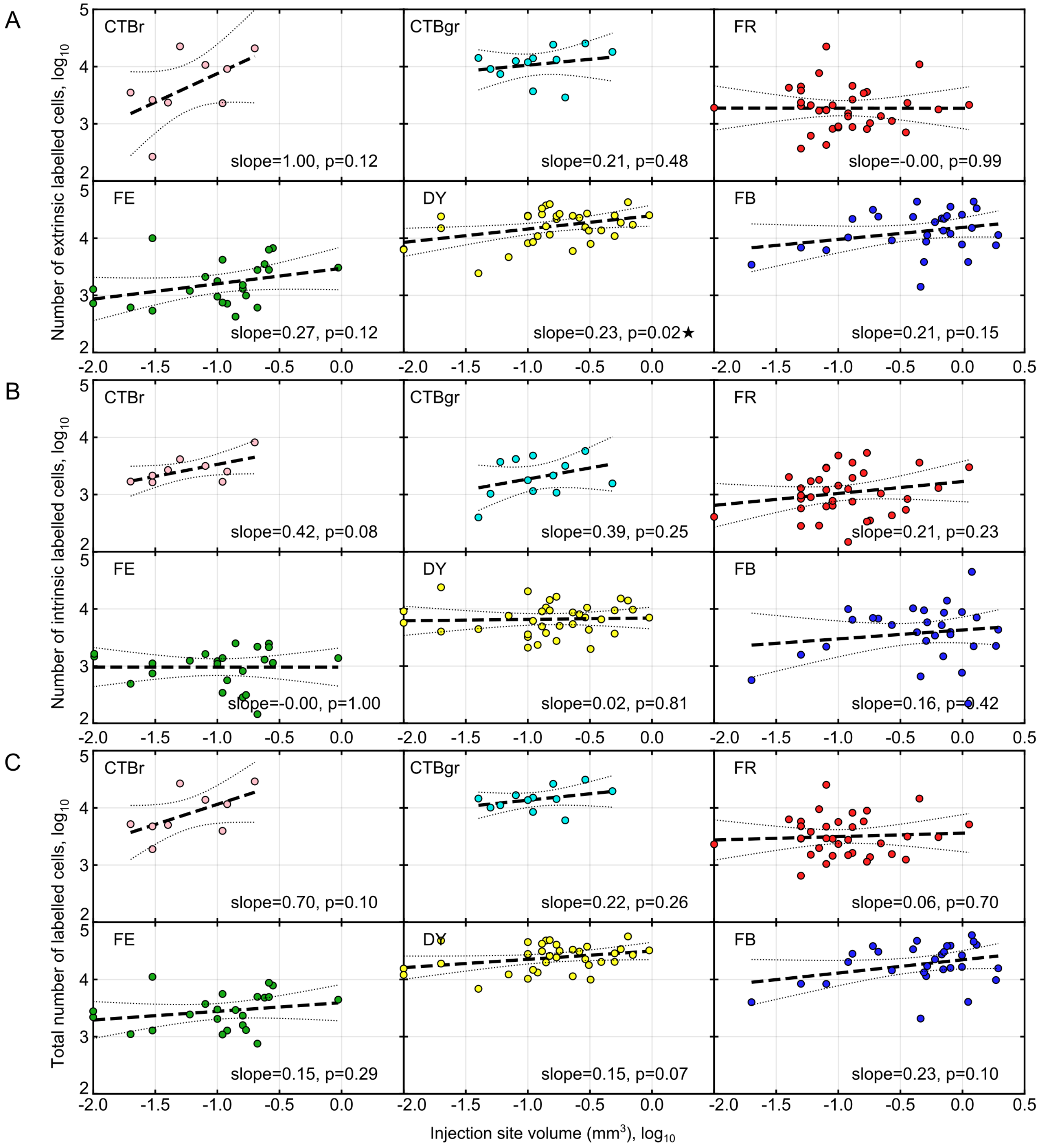

Supplementary Figure 2. Relationship between the number of labeled neurons and injection site volume (related to *Methods*, Surgical procedures). Both quantities are $\log_{10}$-transformed to mitigate the heteroskedasticity. **A**: extrinsic connections; **B**: intrinsic connections; **C**: all labeled neurons. Each panel represents the regression line (thick, dashed line) and the 95% confidence intervals for the regression slopes (dotted lines). No significant relationship was detected, except for the number of extrinsic neurons as a function of DY injection volume (indicated with a star sign). Source data are provided as a Source Data file.

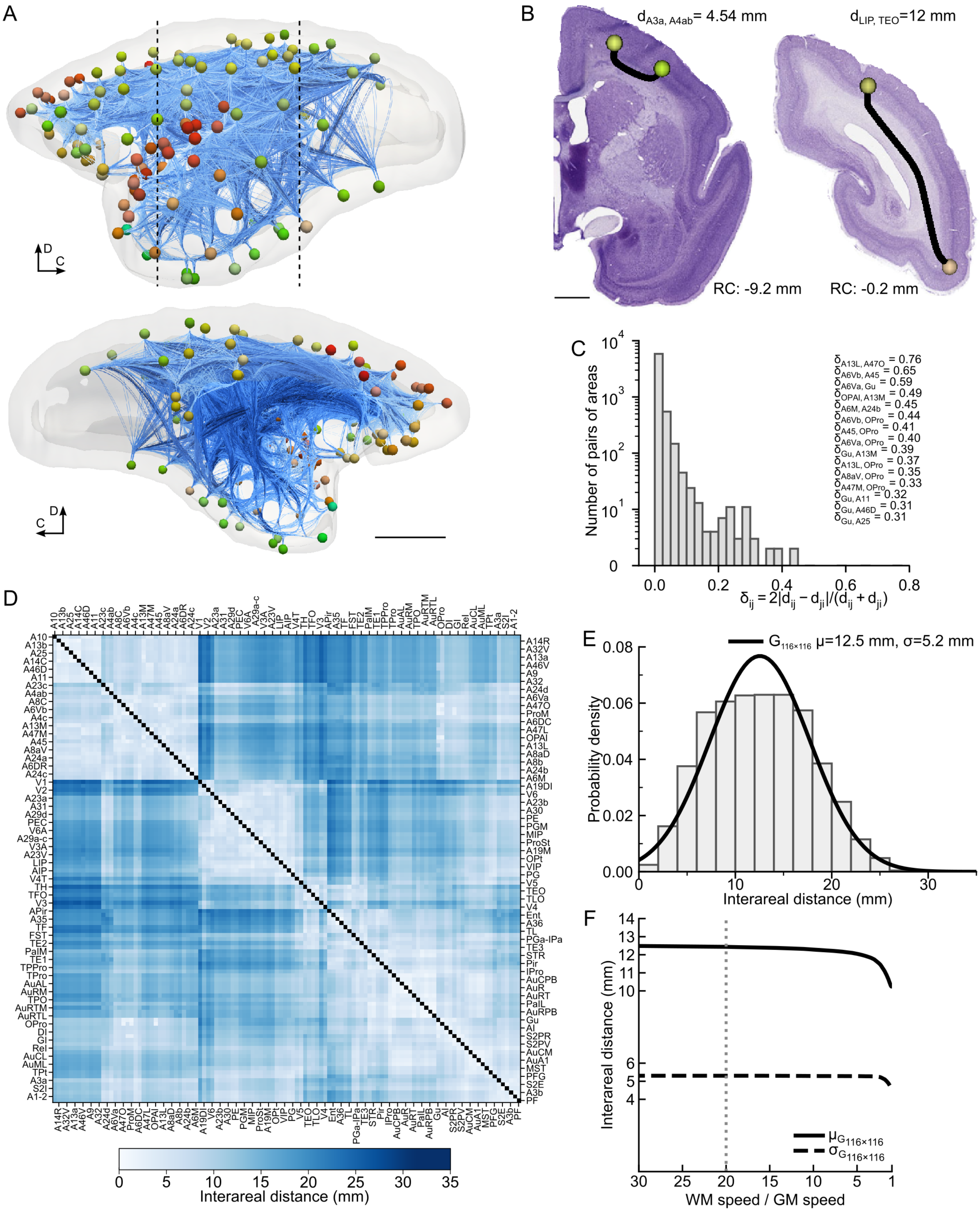

Supplementary Figure 3. Legend provided on the next page.

Legend for the Supplementary Figure 3: Calculation of geodesic paths and interareal distances (related to *Methods*, Estimation of interareal distances using simulated axonal tracts). **A**: lateral (top) and medial (bottom) projections of the visualizations of simulated axonal tracts (curves in different shades of blue) connecting the centroids of cortical areas (spheres in various colors). The gray, semi-transparent outline depicts the external surface of the cerebral cortex as defined in the reference atlas. Scale bar: 5 mm. **B**: Coronal sections with examples of generated fiber tracts lying approximately in coronal plane. The simulated axonal tract connecting areas 3b and 4ab (left section, 9.2 mm rostral to the interaural line), which are relatively close to one another, forms a characteristic U-shape, while that between areas LIP and TEO (right section, 0.2 mm rostral from the interaural line) passes through deeper parts of the white matter. Scale bar: 2 mm. **C**: Summary of the discrepancies of the lengths of axonal tracts between the centroids of cortical areas calculated in the forward direction (i.e. from area i to j) and in the reverse direction (i.e. from area j to i). In the majority of cases, the discrepancies are minor. Pairs of areas with considerable discrepancies are listed next to the histogram. **D**: Interareal distance matrix (available for download through the http://www.marmosetbrain.org portal). Areas are arranged according to hierarchical clustering so that those close to each other are grouped. For details, see *Methods*, 3D reconstruction and mapping procedure. **E**: Histogram of the interareal distances and fitted Gaussian curve. **F**: Comparison of means and standard deviations of distributions of interareal distances obtained with various white matter to gray matter speed ratios (f_{WM} / f_{GM}). The results are stable with only a slight decline of the mean distance as the traversal speed in both tissue classes becomes comparable. The dashed gray line indicates the ratio used in the present resource. The abbreviations of areas are provided in Supplementary Table 1. Source data are provided as a Source Data file.

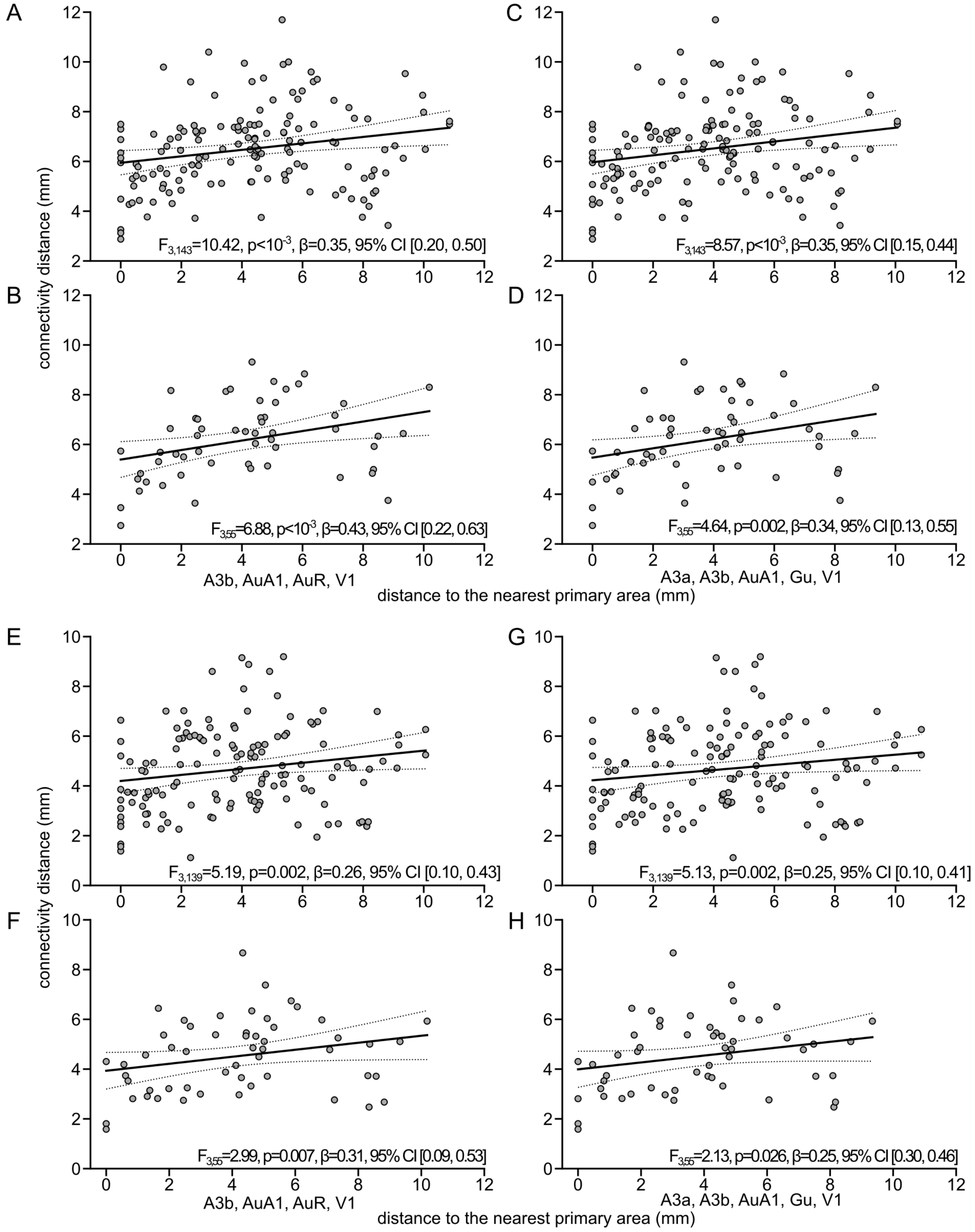

Supplementary Figure 4. Comparison of connectivity distance analysis using different definitions of primary areas (related to Fig. 5). Average afferent connection length scales with distance from the borders of primary sensory areas, whether these are defined by histology or function. **A**, **B**: Analyses in which the locations of individual labeled cells are approximated by locations of centroids of the corresponding areas, based on distance from sensory koniocortex. The average connection length increases with distance from the borders of primary areas whether the analysis is based on individual injections (**A**) or on the averages of the results of injections in the same area (**B**). **C**, **D**: Analogous analyses, using distance from functionally defined primary sensory areas (A3a, A3b: proprioception and tactile somatic sensation; AuA1: hearing; Gu: gustation; V1: vision). **E**-**H**: Analyses in which the stereotaxic coordinates of individual neurons are used to calculate distances to the injection sites. In **E** and **G**, each point represents data from one injection; in **F** and **H**, points represent average values for injections in an area. Source data are provided as a Source Data file.

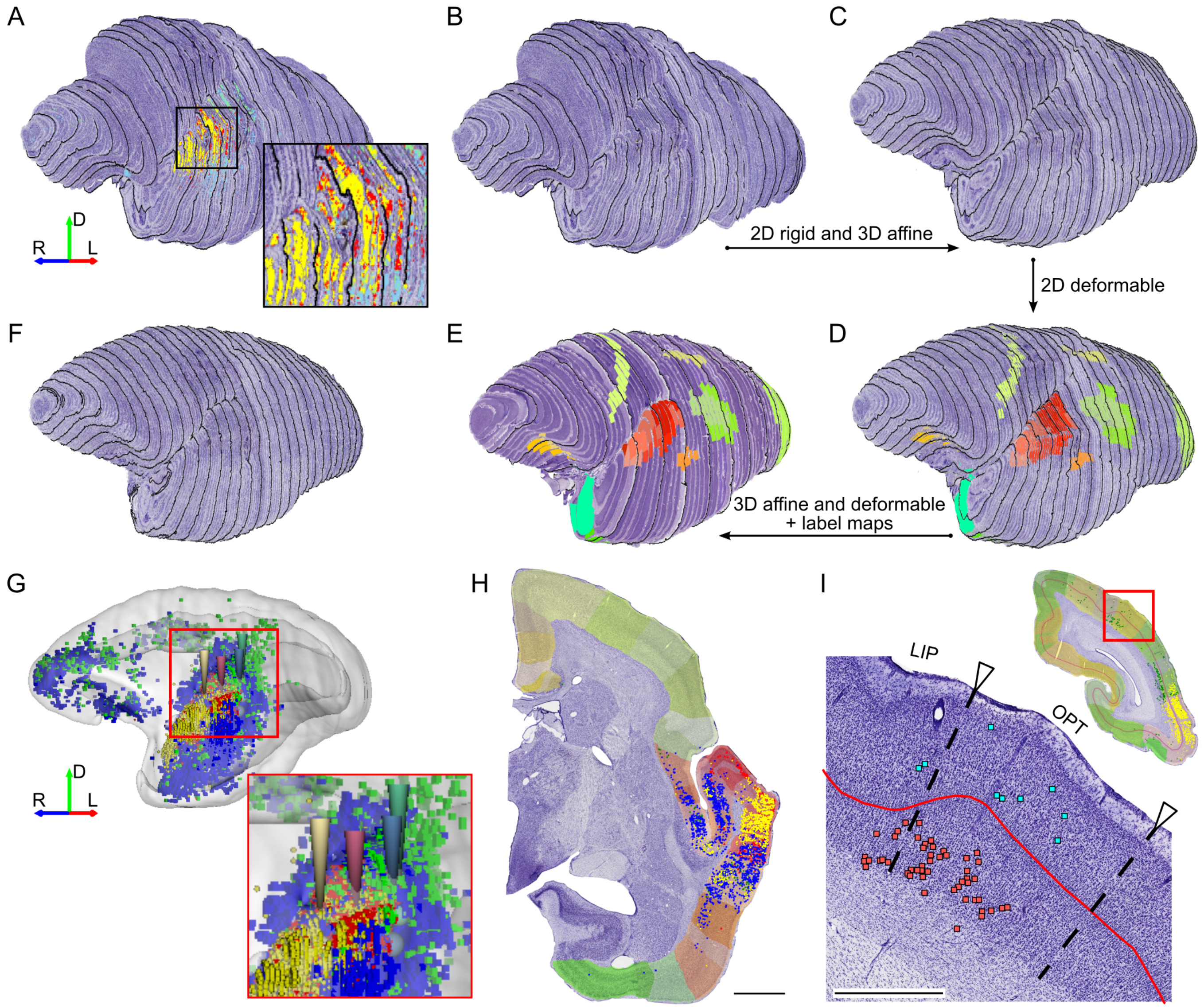

Supplementary Figure 5. Overview of the computational pipeline for mapping the results of the injections into stereotaxic coordinates and for obtaining quantitative connectivity patterns illustrated using an example case (CJ122), Related to *Methods*, 3D reconstruction and mapping procedure. **A**-**G**: Rostrolateral views of a marmoset brain hemisphere undergoing the mapping procedure. Black outlines on every fifth section are provided to better illustrate the general shape of the image stack and are unrelated to the reconstruction process. **A**: alignment of the labeled cells plotted on the fluorescent sections to adjacent Nissl sections. Individual points of different colors correspond to cells labeled by different tracers. The inset presents the vicinity of the DY injection site located in the rostrotemporal auditory area (AuRT). **B**: A stack of 157 Nissl-stained sections ordered rostrocaudally, but not aligned, constitutes the input dataset for the 3D reconstruction. **C**: The stack after the affine reconstruction step which recovers the overall anatomical shape of an individual's brain hemisphere. **D**: The reconstruction after the deformable corrections applied to remove distortions. Different colors represent cytoarchitectural areas delineated manually in this case, to guide the coregistration with the template (see Supplementary Figure 6 for details). In addition, the entire cerebral cortex was outlined as an additional constraint. **E**: 3D visualization of the brain template[2] which constitutes the spatial reference in this study. **F**: The experimental case coregistered to the reference template. Note the similarity to the template in the overall shape and in details. **G**: 3D visualization of the cell mapping results. Points represent individual cells mapped into the stereotaxic space. The tips of the large vertical cones (better visible on the inset) denote the center of mass of individual injections. The gray, semi-transparent outline depicts the external surface of the cerebral cortex. **H**: An example section (7.7 mm rostral to the interaural line) with the parcellation of the cerebral cortex transferred from the reference atlas by the means of registration (color, semi-transparent regions) and labeled cells (color points). Scale bar: 2 mm. **I**: A section (CJ182, 0.2 mm caudal to the interaural line) illustrating the process of dividing the labeled cells into infra- (red points) and supragranular (cyan points). The red curve represents the middle of the layer 4 drawn on sections containing labeled neurons. OPT and LIP are cytoarchitectural areas of the parietal lobe[2] as reconstructed in this individual. Further technical aspects of the reconstruction process have been described previously[3]. Scale bar: 1 mm.

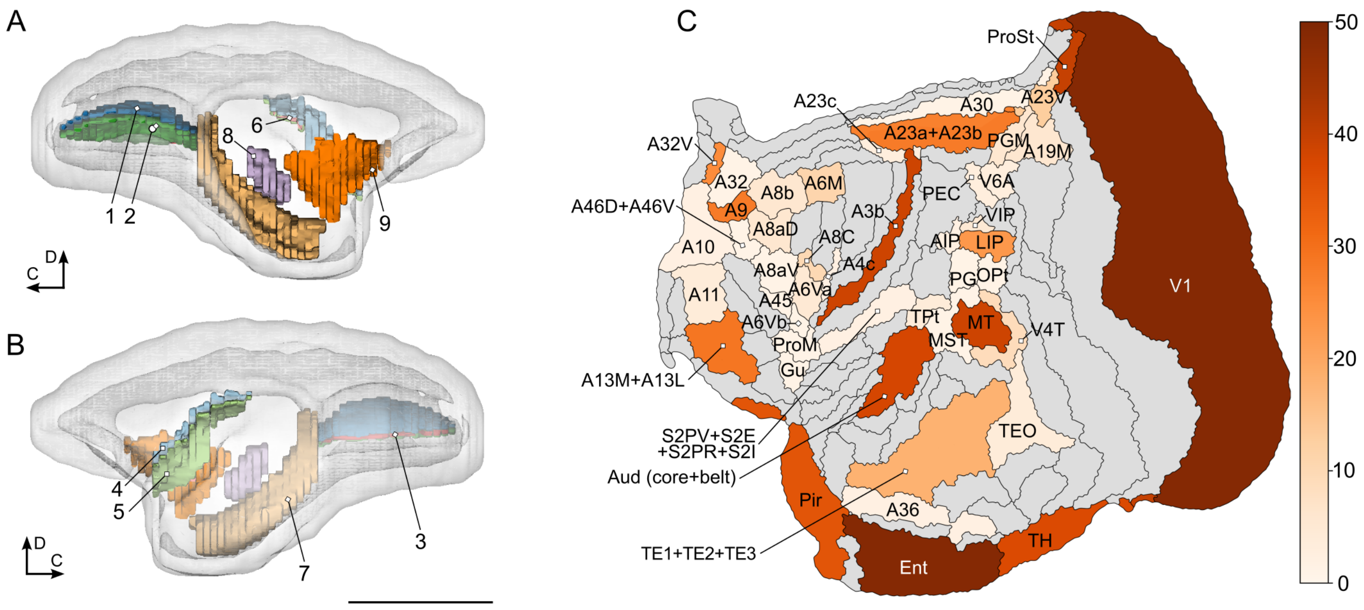

Supplementary Figure 6. Summary of the label maps delineated in both experimental cases as well as in the template, to increase the coregistration accuracy in the expert-assisted reconstruction step (related to *Methods*, Expert-assisted registration to cortical areas). **A**,**B**: Medial and lateral views of the brain outline (gray, transparent model) with major morphological features of the brain shown in different colors: dorsal (1) and ventral (2) banks and the fundus (3) of the calcarine sulcus, medial (4) and lateral (5) banks and the fundus (6) of the lateral sulcus, hippocampus (7), lateral geniculate nucleus (8), and claustrum (9). Scale bar: 10 mm. **C**: Unfolded reconstruction of the cortex (left hemisphere representation; rostral to the left, medial to the top) illustrating how many times, for the entire process of reconstructing all 53 hemispheres, individual cytoarchitectural areas were used to guide the registration process. See Supplementary Table 1 for abbreviations of the areas. Source data are provided as a Source Data file.

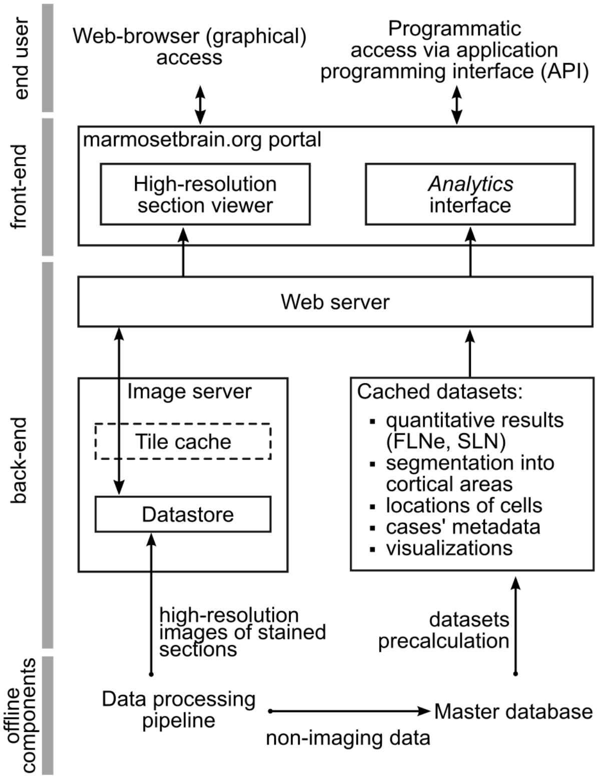

Supplementary Figure 7. The architecture of the http://www.marmosetbrain.org portal (related to *Methods*, Online Portal).

Abbrev.	Full Name
A1/2	areas 1 and 2 of cortex
A10	area 10 of cortex
A11	area 11 of cortex
A13L	area 13 of cortex lateral part
A13M	area 13 of cortex medial part
A13a	area 13a of cortex
A13b	area 13b of cortex
A14C	area 14 of cortex caudal part
A14R	area 14 of cortex rostral part
A19DI	area 19 of cortex dorsointermediate part
A19M	area 19 of cortex medial part
A23V	area 23 of cortex ventral part
A23a	area 23a of cortex
A23b	area 23b of cortex
A23c	area 23c of cortex
A24a	area 24a of cortex
A24b	area 24b of cortex
A24c	area 24c of cortex
A24d	area 24d of cortex
A25	area 25 of cortex
A29a-c	area 29a-c of cortex
A29d	area 29d of cortex
A30	area 30 of cortex
A31	area 31 of cortex
A32	area 32 of cortex
A32V	area 32 of cortex ventral part
A35	area 35 of cortex
A36	area 36 of cortex
A3a	area 3a of cortex (somatosensory)
A3b	area 3b of cortex (somatosensory)
A45	area 45 of cortex
A46D	area 46 of cortex dorsal part
A46V	area 46 of cortex ventral part
A47L	area 47 (old 12) of cortex lateral part
A47M	area 47 (old 12) of cortex medial part
A47O	area 47 (old 12) of cortex orbital part
A4ab	area 4 of cortex parts a and b (primary motor)
A4c	area 4 of cortex part c (primary motor)
A6DC	area 6 of cortex dorsocaudal part
A6DR	area 6 of cortex dorsorostral part
A6M	area 6 of cortex medial (supplementary motor) part
A6Va	area 6 of cortex ventral part a
A6Vb	area 6 of cortex ventral part b
A8C	area 8 of cortex caudal part
A8aD	area 8a of cortex dorsal part
A8aV	area 8a of cortex ventral par
A8b	area 8b of cortex
A9	area 9 of cortex
AI	agranular insular cortex
AIP	anterior intraparietal area of cortex
APir	amygdalopiriform transition area
AuA1	auditory cortex primary area
AuAL	auditory cortex anterolateral area
AuCL	auditory cortex caudolateral area
AuCM	auditory cortex caudomedial area
AuCPB	auditory cortex caudal parabelt area
AuML	auditory cortex middle lateral area
AuR	auditory cortex rostral area
AuRM	auditory cortex rostromedial area
AuRPB	auditory cortex rostral parabelt
AuRT	auditory cortex rostrotemporal
AuRTL	auditory cortex rostrotemporal lateral area
AuRTM	auditory cortex rostrotemporal medial area
DI	dysgranular insular cortex
Ent	entorhinal cortex
FST	fundus of superior temporal sulcus area of cortex
GI	granular insular cortex
Gu	gustatory cortex
IPro	insular proisocortex
LIP	lateral intraparietal area of cortex
MIP	medial intraparietal area of cortex
MST	medial superior temporal area of cortex
OPAl	orbital periallocortex
OPro	orbital proisocortex
OPt	occipito-parietal transitional area of cortex
PE	parietal area PE
PEC	parietal area PE caudal part
PF	parietal area PF (cortex)
PFG	parietal area PFG (cortex)
PG	parietal area PG
PGM	parietal area PG medial part (cortex)
PGa/IPa	Area PGa andIPa (fundus of superior temporal ventral area)
PaIL	parainsular cortex lateral part
PaIM	parainsular cortex medial part
Pir	piriform cortex
ProM	proisocortical motor region (precentral opercular cortex)
ProSt	prostriate area
ReI	retroinsular area (cortex)
S2E	secondary somatosensory cortex external part
S2I	secondary somatosensory cortex internal part
S2PR	secondary somatosensory cortex parietal rostral area
S2PV	secondary somatosensory cortex parietal ventral area
STR	superior temporal rostral area (cortex)
TE1	temporal area TE1 (inferior temporal cortex)
TE2	temporal area TE2 (inferior temporal cortex)
TE3	temporal area TE3 (inferior temporal cortex)
TEO	temporal area TE occipital part
TF	temporal area TF
TFO	temporal area TF occipital part
TH	temporal area TH
TL	temporal area TL
TLO	temporal area TL occipital part
TPO	temporo-parieto-occipital association area (superior temporal polysensory cortex)
TPPro	temporopolar proisocortex
TPro	temporal proisocortex
TPt	temporoparietal transitional area
V1	primary visual cortex
V2	visual area 2
V3	visual area 3 (ventrolateral posterior area)
V3A	visual area 3A (dorsoanterior area)
V4	visual area 4 (ventrolatereral anterior area)
V4T	visual area 4 transitional part (middle temporal crescent)
V5	visual area 5 (middle temporal area)
V6	visual area 6 (dorsomedial area)
V6A	visual area 6A (posterior parietal medial area)
VIP	ventral intraparietal area of cortex

Supplementary Table 1. Abbreviations of the names of cortical areas

Supplementary Table 2: Key materials and their sources

REAGENT or RESOURCE	SOURCE	IDENTIFIER
Chemicals, Peptides, and Recombinant Proteins		
Diamidino Yellow hydrochloride (DY)	Polysciences Inc	CAS 87397-07-7
Fast Blue (FB)	Polysciences Inc	CAS 74749-42-1
Dextran Tetramethylrhodamine 10,000 MW (Fluoro Ruby, FR)	Life Technologies	D1817
Dextran Fluorescein 10,000 MW (Fluoro Emerald, FE)	Life Technologies	D1820
Cholera Toxin Subunit B (Recombinant), Alexa Fluor™ 488 Conjugate (CTBgr)	Life Technologies	C22841
Cholera Toxin Subunit B (Recombinant), Alexa Fluor™ 594 Conjugate (CTBr)	Life Technologies	C22842
Deposited Data		
Release 1.0 of the Marmoset Brain Connectivity Atlas which includes the results of 143 injections of retrograde tracers	This paper	http://www.marmosetbrain.org<br>RRID:SCR_015964
Volumetric digital common marmoset brain template	Majka et al. (2016)	http://r.marmosetbrain.org/marmoset_brain_template.zip
Experimental Models: Organisms/Strains		
Marmoset monkeys	National Non-human Primate Breeding and Research Facility, Australia	
Software and Algorithms		
Possum 3D reconstruction framework v. 0.9.3	Majka and Wójcik (2015)	https://github.com/pmajka/poSSum
Advanced Normalization Tools (ANTS) v. 2.1.0	Klein et al. (2009),<br>Avants et al. (2011)	http://picsl.upenn.edu/software/ants/<br>RRID:SCR_004757
3d Brain Atlas Reconstructor	Majka et al. (2012)	https://github.com/pmajka/3dbar<br>http://service.3dbar.org
Simple ITK v. 1.0.1	Kitware	https://github.com/SimpleITK/SimpleITK
Source code of the Marmoset Brain Connectivity Atlas High-Resolution Section Viewer (http://www.marmosetbrain.org)	This paper	https://github.com/Neuroinflab/marmosetbrain.org
Source code of the interface for exploration of the quantitative results of retrograde tracer injections (http://analysis.marmosetbrain.org)	This paper	https://github.com/Neuroinflab/analysis.marmosetbrain.org
Marmoset Brain Connectivity Atlas Application Programming Interface (API)	This paper	http://analytics.marmosetbrain.org/wiki/api
Other		
PDF version of the book "The Marmoset Brain in Stereotaxic Coordinates" by Paxinos, Watson, Petrides, Rosa and Tokuno	Paxinos et al. (2012)	http://r.marmosetbrain.org/Atlas+Small.pdf
Protocol for Nissl staining of marmoset brain sections	Katrina Worthy and Kathleen Burman, Monash University	http://r.marmosetbrain.org/NisslStain.pdf
Protocol for myelin staining of marmoset brain sections using the Gallyas method	Katrina Worthy, Kathleen Burman and Marcello Rosa, Monash University	http://r.marmosetbrain.org/Gallyasmethod.pdf
Protocol for cytochrome oxidase staining	Katrina Worthy and Marcello Rosa, Monash University	http://r.marmosetbrain.org/CytochromeOxidase.pdf

Supplementary Table 3: Full list of injections.

#	Case	Tracer	Hemisphere Injected	Area(s) involved	Layers involved	Assigned area	Injection site volume (mm^3)	Stereotaxic coordinates (mm)			section	Number of labelled neurons		
								Mediolateral	Rostrocaudal	Dorsoventral		Total	Extrinsic	Intrinsic
1.	CJ19	FE	L	V3a (100%)	1-6	■ V3a	0.28	3.8	+1.4	15.7	m6a	7,848	6,706	1,142
2.	CJ19	FR	L	V2 (100%)	1-5	■ V2	0.17	6.3	+5.9	14.2	m11c	8,951	3,608	5,343
3.	CJ21	FB	L	V3a (72%), V6 (22%), LIP (6%)	1-5	□ V3a	1.11	5.0	+1.6	15.9	9b	3,914	3,693	221
4.	CJ21	DY	L	A19DI (96%), V6 (4%)	1-5	⬓ A19DI	0.32	6.6	+3.2	15.0	12c	9,572	7,579	1,993
5.	CJ36	FR	L	V2 (100%)	1-6	■ V2	0.45	1.0	+4.9	15.7	s3a	14,559	10,948	3,611
6.	CJ36	FB	L	V6 (100%)	1-6	■ V6	1.86	3.2	+3.4	15.9	s9a	9,772	7,518	2,254
7.	CJ50	DY	L	PG (79%), OPt (21%)	1-6	□ PG	0.23	7.2	-1.4	14.7	c26b	33,246	24,572	8,674
8.	CJ50	FR	L	V4 (100%)	1-5	■ V4	0.07	8.3	+0.4	13.7	c23a	9,508	7,701	1,807
9.	CJ51	DY	L	MST (92%), OPt (8%)	1-6	⬓ MST	0.29	8.3	-1.9	13.7	c20b	22,642	15,539	7,103
10.	CJ52	DY	L	PEC (97%), PE (3%)	1-6	⬓ PEC	0.50	2.9	-1.3	16.7	c17a	28,615	24,918	3,697
11.	CJ52	FR	L	V6 (100%)	1-6	■ V6	0.08	4.1	+2.3	16.1	c11b	4,701	1,749	2,952
12.	CJ52	FE	L	V6 (100%)	1-6	■ V6	0.08	2.1	+2.9	16.9	c10b	3,729	2,105	1,624
13.	CJ55	DY	L	PG (97%), MST (3%)	1-6	⬓ PG	0.64	7.3	-1.8	13.6	c24b	56,624	42,594	14,030
14.	CJ55	FR	L	LIP (100%)	1-4	■ LIP	0.08	5.9	-1.5	15.5	c24a	1,045	426	619
15.	CJ55	FB	L	OPt (67%), PG (21%), MST (12%)	1-6	□ OPt	0.60	8.1	-0.9	13.9	c22a	11,251	9,539	1,712
16.	CJ55	FE	L	MIP (59%), V3a (41%)	1-5	□ MIP	0.16	3.2	+0.1	17.0	c21a	1,589	1,305	284
17.	CJ56	FR	L	V5 (100%)	1-5	■ V5	0.18	9.2	-3.1	11.9	c24a	1,378	1,027	351
18.	CJ56	FB	L	V5 (100%)	1-6	■ V5	0.99	9.0	-2.1	12.1	c21a	16,566	7,760	8,806
19.	CJ56	DY	L	V4T (85%), V5 (15%)	1-4	⬓ V4T	0.12	10.7	-1.9	10.6	c20b	13,227	10,881	2,346
20.	CJ56	FE	L	V5 (100%)	1-6	■ V5	0.24	9.8	-0.9	11.0	c18a	4,811	3,512	1,299

21.	CJ64	FE	R	AuML (83%), AuAL (17%)	1-5	⬓ AuML	0.17	10.5	-7.1	9.1	c29a	652	496	156
22.	CJ64	FR	R	AuCM (91%), AuA1 (9%)	1-5	⬓ AuCM	0.17	9.1	-5.7	11.2	c23a	572	404	168
23.	CJ64	FB	R	V4T (81%), V5 (19%)	1-6	⬓ V4T	0.70	10.4	-1.2	10.0	c14b	7,515	6,774	741
24.	CJ70	DY	R	A10 (100%)	1-6	■ A10	0.23	1.4	-19.9	11.8	r2c	11,376	5,955	5,421
25.	CJ70	FB	R	A9 (100%)	1-5	■ A9	1.94	1.0	-16.9	13.2	r8a	15,672	11,329	4,343
26.	CJ70	FR	R	A8aD (100%)	1-5	■ A8aD	0.13	4.1	-16.0	13.6	r10a	1,629	877	752
27.	CJ71	DY	R	A10 (100%)	1-5	■ A10	0.39	1.5	-19.6	10.9	r2b	20,271	13,696	6,575
28.	CJ71	FB	R	A10 (100%)	1-5	■ A10	0.51	2.8	-19.3	11.2	r2d	11,457	8,699	2,758
29.	CJ71	FR	R	A47L (99%), A47M (1%)	1-6	⬓ A47L	0.64	5.2	-17.2	11.5	r5b	3,078	1,778	1,300
30.	CJ73	FR	R	A10 (100%)	2-6	■ A10	1.13	1.1	-18.3	10.9	r4b	2,569	1,068	1,501
31.	CJ73	FB	R	A47L (95%), A8aV(5%)	1-6	⬓ A47L	0.68	5.8	-17.1	12.4	r7b	13,687	11,082	2,605
32.	CJ73	DY	R	A8b (100%)	1-4	■ A8b	0.50	0.4	-15.4	14.6	r10b	10,161	5,438	4,723
33.	CJ73	FE	R	A47L (100%)	1-5	■ A47L	0.94	8.0	-15.9	10.8	r11b	2,217	1,527	690
34.	CJ74	FR	R	A8aV (82%), A47L (18%)	1-6	⬓ A8aV	0.04	6.3	-16.0	12.2	r10b	3,146	2,136	1,010
35.	CJ74	FB	R	A8b (100%)	1-6	■ A8b	0.46	1.4	-15.3	14.2	r12b	1,034	704	330
36.	CJ74	DY	R	A8b (94%), A24c (6%)	1-3	⬓ A8b	0.10	0.2	-13.3	15.0	r17b	5,142	4,091	1,051
37.	CJ75	DY	L	A8aV (82%), A45 (18%)	1-6	⬓ A8aV	0.31	7.4	-14.9	12.0	r14b	17,897	13,575	4,322
38.	CJ75	FR	L	AuA1 (100%)	1-6	■ AuA1	0.12	10.7	-6.6	10.7	r34b	2,767	1,523	1,244
39.	CJ76	FE	L	PFG (100%)	1-5	■ PFG	0.10	7.5	-4.7	14.6	c39b	1,496	887	609
40.	CJ76	DY	L	PFG (97%), AIP (3%)	1-6	⬓ PFG	0.95	6.9	-3.7	15.0	c36b	16,142	12,621	3,521

41.	CJ76	FR	L	PG (100%)	1-6	■ PG	0.35	7.2	-1.4	14.7	c30b	623	353	270
42.	CJ76	FB	L	LIP (72%), Opt (28%)	1-5	□ LIP	0.52	6.7	-0.2	15.4	c27b	8,569	5,648	2,921
43.	CJ77	DY	R	PF (85%), PFG (15%)	1-3	⬓ PF	0.13	7.0	-5.5	15.7	c39b	31,067	26,118	4,949
44.	CJ77	FR	R	PG (100%)	1-6	■ PG	0.05	6.7	-2.2	15.6	c31b	650	368	282
45.	CJ77	FB	R	Opt (79%), LIP (21%)	1-5	□ OPt	0.71	7.0	+0.2	15.2	c25b	30,344	21,753	8,591
46.	CJ78	FE	L	A4ab (100%)	2-5	■ A4ab	0.01	4.7	-9.4	15.3	r26b	2,191	723	1,468
47.	CJ78	FB	L	A4ab (100%)	1-4	■ A4ab	0.12	3.2	-8.6	17.1	r28b	20,233	10,286	9,947
48.	CJ78	DY	L	A3a (78%), A4ab (12%), A3b (10%)	1-4	□ A3a	0.02	6.1	-8.7	15.6	r28b	18,986	14,969	4,017
49.	CJ80	FB	R	A23b (75%), A31 (25%)	1-6	□ A23b	0.21	0.5	-1.8	15.5	c36a	30,608	23,845	6,763
50.	CJ80	FR	R	A23a (96%), A23b (4%)	1-4	⬓ A23a	0.09	0.8	-1.4	13.6	c35a	1,452	815	637
51.	CJ80	DY	R	PGM (100%)	1-6	■ PGM	0.14	0.6	-0.3	14.8	c32a	19,929	16,134	3,795
52.	CJ81	FR	L	V2 (63%), V1 (37%)	3-5	□ V2	0.06	0.8	+3.4	13.3	c20b	1,909	1,055	854
53.	CJ81	FB	L	V2 (100%)	1-6	■ V2	0.27	0.7	+4.8	14.4	c17b	7,196	4,577	2,619
54.	CJ81	DY	L	V2 (100%)	1-6	■ V2	0.56	1.9	+6.2	15.9	c14b	16,950	9,348	7,602
55.	CJ81	FE	L	V2 (100%)	1-5	■ V2	0.06	5.9	+6.1	14.7	c14b	1,221	600	621
56.	CJ82	DY	L	V2 (90%), V1 (10%)	1-4	⬓ V2	0.11	0.3	+4.0	13.3	c18a	14,752	8,578	6,174
57.	CJ82	FR	L	V1 (100%)	2-5	■ V1	0.10	0.4	+5.5	13.1	c14b	5,673	859	4,814
58.	CJ82	FE	L	V1 (100%)	3	■ V1	0.14	2.4	+8.4	15.4	c6b	2,924	424	2,500
59.	CJ83	DY	L	A8b (100%)	1-6	■ A8b	0.70	0.9	-14.3	14.4	r13b	13,440	8,574	4,866
60.	CJ84	FE	L	A23a (98%), A30 (2%)	2-3	⬓ A23a	0.02	0.5	-1.7	13.6	c17a	1,104	613	491

61.	CJ84	FB	L	PGM (73%), A23b (27%)	1-4	□ PGM	0.13	0.8	-0.1	12.8	c15a	28,158	21,697	6,461
62.	CJ84	FR	L	A19M (100%)	1-3	■ A19M	0.07	0.7	+0.3	12.6	c14c	1,980	1,694	286
63.	CJ90	FR	R	V2 (100%)	1-6	■ V2	0.13	2.4	+7.0	15.2	c9a	8,215	4,604	3,611
64.	CJ90	DY	R	V2 (100%)	1-3	■ V2	0.02	3.3	+7.0	15.5	c9a	47,948	24,027	23,921
65.	CJ94	DY	R	A8aV (94%), A6Va 6%)	1-6	⬓ A8aV	0.14	6.6	-14.0	12.3	r16b	24,006	18,710	5,296
66.	CJ94	FR	R	A6DR (100%)	1-6	■ A6DR	0.28	4.8	-12.3	14.6	r20b	746	672	74
67.	CJ94	FB	R	A6M (100%)	1-6	■ A6M	0.80	1.3	-11.5	16.0	r21b	7,959	5,985	1,974
68.	CJ100	FR	R	A6DR (100%)	1-6	■ A6DR	0.09	3.4	-12.7	14.6	r17b	2,879	2,105	774
69.	CJ100	FE	R	A6Va (91%), A8C (9%)	1-5	⬓ A6Va	0.26	6.6	-13.2	12.8	r17b	8,765	6,288	2,477
70.	CJ102	DY	L	A3b (86%), A1/2 (14%)	1-6	⬓ **A3b**	0.30	8.9	-10.8	12.0	r12b	37,537	27,016	10,521
71.	CJ108	FE	R	A8aV (95%), A47L (5%)	1-5	⬓ A8aV	0.21	5.8	-16.0	12.7	r10a	4,965	2,784	2,181
72.	CJ108	FR	R	A8aD (100%)	1-6	■ A8aD	0.22	3.9	-15.4	13.7	r11a	2,399	1,359	1,040
73.	CJ110	FE	L	A6DR (100%)	1-6	■ A6DR	0.26	3.8	-12.5	14.6	r17b	4,932	2,795	2,137
74.	CJ110	FR	L	A6Va (100%)	1-5	■ A6Va	0.27	7.5	-13.1	12.2	r17b	1,554	1,122	432
75.	CJ111	FE	L	A6Va (100%)	1-6	■ A6Va	0.16	7.5	-12.0	12.0	r20a	2,334	1,514	820
76.	CJ111	FR	L	A6DC (99%), A4ab (1%)	1-6	⬓ A6DC	0.36	4.1	-11.0	15.3	r21b	3,148	2,312	836
77.	CJ112	FE	L	A6DC (96%), A6M (4%)	1-5	⬓ A6DC	0.12	3.2	-11.5	15.7	r20a	5,578	4,197	1,381
78.	CJ112	FR	L	A6Va (100%)	1-5	■ A6Va	0.05	8.1	-12.1	12.4	r20a	2,890	2,048	842
79.	CJ113	FE	L	A6M (100%)	1-5	■ A6M	0.01	0.7	-12.3	15.8	r19a	2,784	1,277	1,507
80.	CJ113	FR	L	A4ab (100%)	1-6	■ A4ab	0.10	1.0	-9.4	16.8	r26a	2,330	900	1,430

81.	CJ114	FR	R	A4ab (100%)	1-6	■ A4ab	0.16	3.4	-10.5	15.3	r25a	5,796	3,423	2,373
82.	CJ114	FE	R	A3b (100%)	1-5	■ A3b	0.03	8.2	-10.7	11.9	r25a	1,283	539	744
83.	CJ115	FE	R	A6DC (100%)	1-6	■ A6DC	0.10	3.3	-11.8	15.0	r19b	2,042	950	1,092
84.	CJ115	FR	R	A4c (96%), A8C (4%)	1-5	⬓ A4c	0.05	6.9	-11.5	12.4	r20b	2,926	2,354	572
85.	CJ116	FR	R	A6DR (100%)	1-5	■ A6DR	0.08	4.0	-13.2	14.8	r16a	2,926	1,727	1,199
86.	CJ116	FE	R	A6Va (100%)	1-5	■ A6Va	0.12	7.1	-12.7	12.4	r18a	1,279	715	564
87.	CJ122	DY	R	AuRT (63%), AuAL (37%)	1-6	□ AuRT	0.17	10.7	-8.9	8.3	r28b	24,278	21,527	2,751
88.	CJ122	FR	R	AuCPB (90%), AuAL (10%)	1-4	⬓ AuCPB	0.05	10.8	-7.2	7.8	r33a	5,766	4,478	1,288
89.	CJ122	FB	R	TPO (75%), PGa/IPa (20%), AuCPB (5%)	1-5	□ TPO	1.30	10.8	-5.4	8.4	r37b	40,291	33,214	7,077
90.	CJ122	FE	R	TPO (79%, AuCL (21%)	1-3	□ TPO	0.03	10.7	-5.1	9.6	r38b	11,112	9,998	1,114
91.	CJ123	FE	R	A8C (92%), A6Va (8%)	1-5	⬓ A8C	0.21	6.3	-13.1	13.7	r17b	754	610	144
92.	CJ123	FR	R	A6DC (100%)	1-6	■ A6DC	0.06	4.0	-11.3	15.0	r21a	1,516	617	899
93.	CJ125	FR	L	A8aV (100%)	1-5	■ A8aV	0.13	6.6	-15.0	12.7	r14a	4,612	2,657	1,955
94.	CJ125	FE	L	A6DR (100%)	1-6	■ A6DR	0.11	4.0	-13.5	14.6	r17a	1,090	749	341
95.	CJ146L	DY	L	A23b (55%), A23a (45%)	1-6	□ A23b	0.15	1.1	-4.9	14.9	r41a	48,825	39,408	9,417
96.	CJ146L	FR	L	A23a (90%), A23b (6%), A30 (4%)	5-6	⬓ A23a	0.08	1.3	-1.8	14.1	r49a	12,678	11,231	1,447
97.	CJ146	FB	R	A23b (92%), A31 (8%)	1-5	⬓ A23b	0.80	0.3	-3.5	15.8	r45a	19,542	17,757	1,785
98.	CJ148R	DY	L	A32V (100%)	1-5	■ A32V	0.13	0.8	-15.7	9.8	r6a	42,181	33,127	9,054
99.	CJ153	FR	L	A23b (100%)	3-4	■ A23b	0.05	0.6	-2.3	15.2	c17d	4,770	3,807	963
100.	CJ164	DY	L	A8b (100%)	1-4	■ A8b	0.17	0.7	-15.7	13.8	r6d	40,621	24,275	16,346

101.	CJ164	FB	L	A8b (97%), A24c (3%)	1-3	⬓ A8b	0.40	0.2	-14.5	14.3	r8c	33,758	23,502	10,256
102.	CJ164	CTBgr	L	A6M (100%)	1-5	■ A6M	0.20	0.7	-12.9	15.4	r10c	6,060	2,886	3,174
103.	CJ167	CTBgr	R	A24d (93%), A24c (7%)	1-3	⬓ A24d	0.05	0.3	-10.5	15.7	r12a	10,122	9,092	1,030
104.	CJ167	FB	R	A24d (100%)	1-3	■ A24d	0.02	0.6	-9.5	15.8	r13b	4,003	3,435	568
105.	CJ167	FR	R	A24d (72%, A23c (28%)	2-3	□ A24d	0.01	0.8	-8.6	16.0	r14c	2,309	1,903	406
106.	CJ167	DY	R	A23c (69%), A3a (22%), A3a (9%)	2-5	□ A23c	0.18	0.5	-7.7	16.1	r15d	31,613	26,563	5,050
107.	CJ170	DY	R	A6M (100%)	1-5	■ A6M	0.04	0.7	-12.8	15.5	r9d	6,873	2,433	4,440
108.	CJ170	FB	R	A3a (100%)	1-6	■ A3a	0.08	2.5	-7.5	16.4	r15d	8,359	6,171	2,188
109.	CJ170	CTBr	R	A3b (100%)	1-5	■ A3b	0.11	5.8	-7.3	15.4	r15d	3,961	2,291	1,670
110.	CJ170	CTBgr	R	S2E (80%), PF (14%), S2I (6%)	1-6	⬓ S2E	0.48	7.4	-6.1	13.2	r17a	19,648	18,085	1,563
111.	CJ173	DY	R	A4ab (100%)	1-4	■ A4ab	0.01	2.2	-8.9	16.8	r14c	15,544	6,448	9,096
112.	CJ173	FB	R	A1-2 (91%), A3b (7%), and PF (2%)	1-6	⬓ A1/2	0.19	5.5	-6.6	15.2	r18a	38,334	31,389	6,945
113.	CJ173	CTBr	R	PE (63%), A1-2 (37%)	1-6	□ PE	0.02	2.5	-5.3	17.0	r19d	5,197	3,512	1,685
114.	CJ173	CTBgr	R	AIP (55%), PG (45%)	1-6	□ AIP	0.04	5.9	-3.0	15.5	r22d	14,575	14,178	397
115.	CJ174	FB	R	V1 (100%)	1-6	■ V1	1.19	3.2	+8.6	11.1	c5a	59,682	15,148	44,534
116.	CJ174	CTBgr	R	V1 (100%)	1-3	■ V1	0.11	5.9	+10.0	10.2	c3a	8,496	3,697	4,799
117.	CJ174	CTBr	R	V1 (100%)	1-3	■ V1	0.03	1.5	+10.3	13.8	c2d	1,899	265	1,634
118.	CJ178	CTBr	R	A10 (100%)	1-6	■ A10	0.03	2.4	-18.4	12.7	r3a	4,755	2,611	2,144
119.	CJ178	CTBgr	R	A10 (100%)	1-6	■ A10	0.06	3.4	-18.4	12.4	r3a	11,133	7,418	3,715
120.	CJ178	DY	R	A32 (97%), A9 (3%)	2-5	⬓ A32V	0.07	0.9	-16.1	11.6	r6a	12,267	4,664	7,603

121.	CJ178	FB	R	V1 (100%)	1-6	■ V1	0.49	3.3	+7.3	12.8	c6d	13,301	3,837	9,464
122.	CJ180	FB	R	AuRT (81%), AuRTL (11%), AuAL (8%)	1-6	⬓ AuRT	0.99	10.5	-9.4	8.2	r13b	26,438	25,672	766
123.	CJ180	CTBr	R	TE3 (100%)	1-4	■ TE3	0.20	10.6	-6.2	5.2	r16c	29,029	20,867	8,162
124.	CJ180	CTBgr	R	AuML (77%), AuA1 (23%)	1-6	□ AuML	0.11	10.4	-5.9	10.1	r17d	15,109	13,961	1,148
125.	CJ180	DY	R	TE3 (100%)	1-5	■ TE3	0.15	11.0	-3.9	6.1	r20a	25,870	11,530	14,340
126.	CJ181	CTBr	R	A11 (100%)	1-6	■ A11	0.05	3.5	-17.5	11.0	r3d	26,736	22,598	4,138
127.	CJ181	DY	R	A47L (95%), A46V (3%), A8aV (2%)	1-6	⬓ A47L	0.26	5.4	-17.0	12.0	r4c	30,799	22,724	8,075
128.	CJ181	CTBgr	R	AuCM (64%), MST (27%), AuA1 (9%)	1-6	□ AuCM	0.29	9.5	-4.8	11.7	c21c	31,190	25,416	5,774
129.	CJ181	FB	R	TEO (100%)	1-5	■ TEO	0.75	10.9	+0.3	4.5	c14d	38,394	24,422	13,972
130.	CJ182	CTBr	R	PGa/ IPa (100%)	1-3	■ PGa/IPa	0.12	10.5	-5.4	7.2	r18d	11,626	9,106	2,520
131.	CJ182	CTBgr	R	V4 (88%), V4T (12%)	1-4	⬓ V4	0.08	10.0	+1.7	9.5	c13b	16,665	12,489	4,176
132.	CJ182	DY	R	V4 (100%)	1-5	■ V4	0.10	10.4	+1.8	7.4	c13a	45,148	24,780	20,368
133.	CJ800	DY	R	A46D (100%)	1-5	■ A46D	0.01	3.3	-17.5	12.6	r8a	12,038	6,324	5,714
134.	CJ800	FB	R	A47L (90%), A46V (10%)	1-3	⬓ A47L	0.05	5.7	-16.9	13.1	r9b	8,414	6,836	1,578
135.	CJ800	CTBr	R	A8aD (100%)	1-6	■ A8aD	0.04	3.6	-16.1	13.1	r12a	5,029	2,348	2,681
136.	CJ800	CTBgr	R	A45 (67%), A47L (33%)	1-5	⬓ A45	0.16	7.5	-14.5	11.1	r16a	26,385	24,235	2,150
137.	CJ801	DY	R	A46D (100%)	1-6	■ A46D	0.10	2.9	-17.3	13.0	r4d	27,249	24,013	3,236
138.	CJ801	CTBgr	R	A6DR (95%), A8aV (5%)	1-6	⬓ A6DR	0.10	5.0	-14.1	13.5	r8b	13,718	11,938	1,780
139.	CJ801	FB	R	A8C (68%), A6DC (14%), A6Va (11%),	1-6	□ A8C	1.23	6.1	-12.7	13.6	r9c	45,854	43,632	2,222
140.	CJ802	CTBr	R	S2E (100%)	1-3	■ S2E	0.08	8.8	-7.1	12.7	r16c	13,858	10,697	3,161

141.	CJ802	CTBgr	R	AuML (73%), AuCL (11%), AuA1 (9%),	1-6	□ AuML	0.17	10.8	-5.9	10.3	r17d	14,288	13,211	1,077
142.	CJ802	DY	R	AIP (80%), PFG (20%)	1-5	⬓ AIP	0.10	6.0	-3.9	15.6	r20d	27,933	24,346	3,587
143.	CJ802	FB	R	PG (36%), MST (28%), PFG (27%), TPT (8%)	1-5	□ PG	0.43	8.0	-3.2	13.7	r21b	47,485	43,574	3,911

Tracers	
DY	Diamidino Yellow
FE	Fluoro-emerald
FR	Fluoro-ruby
FB	Fast blue
CTBgr	Cholera Toxin Subunit B, 488nm
CTBr	Cholera Toxin Subunit B, red

Involvement of adjacent areas
■ Injection confined entirely within a single area.
⬓ ≥ 80% of the injection within a single area. Involvement of additional areas cannot be ruled out.
□ < 80% of the injection within a single area. Invasion of adjacent areas is very likely.

Stereotaxic coordinates		
Mediolateral	Lateral to the midsagittal plane	
Rostrocaudal	Negative	Rostral to interaural line
	Positive	Caudal to interaural line
Dorsoventral	Dorsal to the interaural line	

Supplementary References

1. Condé, F. Further studies on the use of the fluorescent tracers fast blue and diamidino yellow: effective uptake area and cellular storage sites. *J. Neurosci. Methods* **21**, 31–43, (1987).
2. Paxinos, G., Watson, C., Petrides, M., Rosa, M. & Tokuno, H. *The marmoset brain in stereotaxic coordinates* (Academic Press, 2012), 1st ed.
3. Majka, P. *et al.* Towards a comprehensive atlas of cortical connections in a primate brain: Mapping tracer injection studies of the common marmoset into a reference digital template. *J. Comp. Neurol.* **524**, 2161–2181, (2016).
