# Supplementary material for: Open access resource for cellular-resolution analyses of corticocortical connectivity in the marmoset monkey
Source: Nat Commun. 2020 Feb 28;11:1133. doi: 10.1038/s41467-020-14858-0 (PMC7048793; doi:10.1038/s41467-020-14858-0)
Supplement: Supplementary file 3 — Reporting Summary [file 41467_2020_14858_MOESM3_ESM.pdf]

nature research

Corresponding author(s): Marcello Rosa <marcello.rosa@monash.edu>

Last updated by author(s): Dec 21, 2019

Reporting Summary

Nature Research wishes to improve the reproducibility of the work that we publish. This form provides structure for consistency and transparency in reporting. For further information on Nature Research policies, see Authors & Referees and the Editorial Policy Checklist .

Statistics

For all statistical analyses, confirm that the following items are present in the figure legend, table legend, main text, or Methods section.

n/a	Confirmed	
☐	☒	The exact sample size (n) for each experimental group/condition, given as a discrete number and unit of measurement
☒	☐	A statement on whether measurements were taken from distinct samples or whether the same sample was measured repeatedly
☐	☒	The statistical test(s) used AND whether they are one- or two-sided<br>*Only common tests should be described solely by name; describe more complex techniques in the Methods section.*
☐	☒	A description of all covariates tested
☒	☐	A description of any assumptions or corrections, such as tests of normality and adjustment for multiple comparisons
☐	☒	A full description of the statistical parameters including central tendency (e.g. means) or other basic estimates (e.g. regression coefficient) AND variation (e.g. standard deviation) or associated estimates of uncertainty (e.g. confidence intervals)
☐	☒	For null hypothesis testing, the test statistic (e.g. F, t, r) with confidence intervals, effect sizes, degrees of freedom and P value noted<br>*Give P values as exact values whenever suitable.*
☒	☐	For Bayesian analysis, information on the choice of priors and Markov chain Monte Carlo settings
☒	☐	For hierarchical and complex designs, identification of the appropriate level for tests and full reporting of outcomes
☐	☒	Estimates of effect sizes (e.g. Cohen's d, Pearson's r), indicating how they were calculated

Our web collection on statistics for biologists contains articles on many of the points above.

Software and code

Policy information about availability of computer code

Data collection	The data were acquired using Aperio Scanscope AT Turbo commercial slide scanning system (Leica Biosystems) with a properietary Leica ePathology Solutions v102.0.7.5 software and further compressed into JP2000 format with the Kakadu SDK library v7.3.1 (kakadusoftware.com). The fluorescent labelled cells were plotted with the help of a digitizing system controlled by MDPLOT 5.2 software (Accustage).
Data analysis	The data analysis followed the steps detailed in the Online Methods sections. In brief, Advanced Normalization Tools (ANTS, 1.9, http://picsl.upenn.edu/software/ants), Possum 3D reconstruction framework (v0.9.8, https://github.com/pmajka/poSSum) and the 3d Brain Atlas Reconstructor (https://github.com/pmajka/3dbar) were used to map the experimental data into the reference template. Miscellaneous image processing tasks were carried out using the SimpleITK suite (http://www.simpleitk.org/). The Online portal relies primarily on Djatoka image server v1.1 (https://www.osti.gov/biblio/960585), Celery v4.2 task queue for the back-end, and OpenLayers v4.6 (https://openlayers.org/) combined with ReactJS (v15.4.2) framework for the graphical user interface. The source code of the portal is released under the MIT license (see: github.com/Neuroinflab/marmosetbrain.org and github.com/Neuroinflab/analysis.marmosetbrain.org).

For manuscripts utilizing custom algorithms or software that are central to the research but not yet described in published literature, software must be made available to editors/reviewers. We strongly encourage code deposition in a community repository (e.g. GitHub). See the Nature Research guidelines for submitting code & software for further information.

Data

Policy information about availability of data

All manuscripts must include a data availability statement. This statement should provide the following information, where applicable:

- Accession codes, unique identifiers, or web links for publicly available datasets
- A list of figures that have associated raw data
- A description of any restrictions on data availability

The cortico-cortical connectivity datasets (RRID:SCR_015964) generated and analyzed in the current study are available under the terms of Creative Commons Attribution-ShareAlike 4.0 License and publicly available through the Marmoset Brain Connectivity Atlas portal (www.marmosetbrain.org).

Field-specific reporting

Please select the one below that is the best fit for your research. If you are not sure, read the appropriate sections before making your selection.

☒ Life sciences ☐ Behavioural & social sciences ☐ Ecological, evolutionary & environmental sciences

For a reference copy of the document with all sections, see nature.com/documents/nr-reporting-summary-flat.pdf

Life sciences study design

All studies must disclose on these points even when the disclosure is negative.

Sample size	143 tracer injections in the cerebral cortex, obtained in 52 marmoset monkeys .
Data exclusions	Some of the injection sites placed in the same animals that form the basis of the Resource were excluded from analysis if they invaded the white matter underlying the cortex. These injection sites were not reported or analyzed
Replication	The objective was to place injection sites across a wide expanse of the cerebral cortex, which collectively sampled a variety of locations. No two injection sites were placed in exactly the same coordinates. When more than one injection was assigned to the same cytoarchitectural area, we report not only the mean result, but also the results of individual experiments.<br>The data collection involving plotting the location of tracer-labeled neurons was performed independently by two investigators, who did not have access to each other's files until the entire procedure was completed for a case.
Randomization	Not relevant here.
Blinding	The investigators who performed the data collection (plotting of locations of labeled neurons in histological sections) remained blind to the assignment of the injection site to a cytoarchitectural area.

Reporting for specific materials, systems and methods

We require information from authors about some types of materials, experimental systems and methods used in many studies. Here, indicate whether each material, system or method listed is relevant to your study. If you are not sure if a list item applies to your research, read the appropriate section before selecting a response.

Materials & experimental systems

n/a	Involved in the study	
☒	☐	Antibodies
☒	☐	Eukaryotic cell lines
☒	☐	Palaeontology
☐	☒	Animals and other organisms
☒	☐	Human research participants
☒	☐	Clinical data

Methods

n/a	Involved in the study	
☒	☐	ChIP-seq
☒	☐	Flow cytometry
☒	☐	MRI-based neuroimaging

Animals and other organisms

Policy information about studies involving animals; ARRIVE guidelines recommended for reporting animal research

Laboratory animals	52 young adult marmoset monkeys, Callithrix jacchus.(1.4–4.6 years, median age: 2.5 years; 31 male, 21 female).
Wild animals	Not applicable, all animals were obtained from a primate breeding centre (National Non-human Primate Breeding and Research Facility, Australia)
Field-collected samples	Not applicable, all samples were obtained in a laboratory from animals bred for scientific purposes.

Ethics oversight	Monash University Animal Experimentation Ethics Committee

Note that full information on the approval of the study protocol must also be provided in the manuscript.
