# Supplementary material for: Open access resource for cellular-resolution analyses of corticocortical connectivity in the marmoset monkey
Source: Nat Commun. 2020 Feb 28;11:1133. doi: 10.1038/s41467-020-14858-0 (PMC7048793; doi:10.1038/s41467-020-14858-0)
Supplement: Supplementary file 4 — Source Code [file 41467_2020_14858_MOESM4_ESM.zip › Source_Code/README.pdf]

Overview

This code was developed to accompany the paper:

OPEN ACCESS RESOURCE FOR CELLULAR-RESOLUTION CORTICOCORTICAL CONNECTIVITY IN THE MARMOSET MONKEY

by Piotr Majka, Shi Bai, Sophia Bakola, Sylwia Bednarek, Jonathan M. Chan, Natalia Jermakow, Lauretta Passarelli, David H. Reser, Panagiota Theodoni, Katrina H. Worthy, Xiao-Jing Wang, Daniel K.Wójcik, Partha P. Mitra, Marcello G.P. Rosa

Its purpose is to enable replication of results presented in the study.

System requirements

Hardware requirements

This code requires a standard desktop computer with sufficient RAM for in-memory operations.

Software requirements

OS requirements

This package was developed for Linux systems, and has been tested on:

- Linux: Ubuntu 16.04.6 LTS

Python dependencies

A stack of scientific libaries for python is required:

- statsmodels
- numpy
- pandas
- seaborn
- matplotlib
- scipy

This software has been tested for following dependencies versions:

- Python 2.7.12
- statsmodels==0.8.0
- numpy==1.11.0
- pandas==0.22
- seaborn==0.8.1
- matplotlib==2.2.4
- scipy==1.3.1

Installation Guide

Fonts

Optional fonts instalation for better looking figures:

```
sudo apt-get install msttcorefonts
```

Dependencies

We recommend installing required packages in a separate virtual environment. Typical install time should less than 5 minutes.

```
pip install -r requirements.txt
```

```
cd /your/dir/of/choice
unzip {filename}.zip
```

Execution time

All computations should complete within a few seconds.

Reproduction instructions

Figure 5: Average afferent connection length scales with distance from the borders of primary areas

Panels A, B

```
cd F5_AB_reproduce/
python 00_scatterplot.py
```

Output:

- gradient_areas_True_total_A3a_A3b_A4ab_A4c_AuA1_AuR_AuRT_V1.png
- gradient_areas_True_total_A3a_A3b_A4ab_A4c_AuA1_AuR_AuRT_V1.svg
- report_per_area_total_AuRT-A4c-V1-A4ab-A3b-A3a-AuR-AuA1.txt
- report_per_injection_total_AuRT-A4c-V1-A4ab-A3b-A3a-AuR-AuA1.txt

Panels C, D, F, G

```
cd F5_CDEFG_reproduce/
```

```
python 00_scatterplot.py
python 01_scatterplot_ratio.py
```

Output:

- gradient_areas_True_ratio_A3a_A3b_A4ab_A4c_AuA1_AuR_AuRT_V1.png
- gradient_areas_True_ratio_A3a_A3b_A4ab_A4c_AuA1_AuR_AuRT_V1.svg
- gradient_areas_True_total_A3a_A3b_A4ab_A4c_AuA1_AuR_AuRT_V1.png
- gradient_areas_True_total_A3a_A3b_A4ab_A4c_AuA1_AuR_AuRT_V1.svg
- report_per_area_infra___A3a_A3b_A4ab_A4c_AuA1_AuR_AuRT_V1.txt
- report_per_area_ratio___A3a_A3b_A4ab_A4c_AuA1_AuR_AuRT_V1.txt
- report_per_area_supra___A3a_A3b_A4ab_A4c_AuA1_AuR_AuRT_V1.txt
- report_per_area_total___A3a_A3b_A4ab_A4c_AuA1_AuR_AuRT_V1.txt
- report_per_injection_ratio___A3a_A3b_A4ab_A4c_AuA1_AuR_AuRT_V1.txt
- report_per_injection_total___A3a_A3b_A4ab_A4c_AuA1_AuR_AuRT_V1.txt

Figure 7: Networks of cortical areas are characterized by different proportions of local versus distant afferent connections

Panel B

```
cd F5_CDEFG_reproduce/
python 00_run_me.py
```

Output:

- ratio.png
- out_fln_ratio_4_8_report.txt

Figure S1: Sample characteristics with respect to the six different tracers used

```
cd S1_reproduce/
chmod +x make.sh
./make.sh
```

Output:

- 00_injections_per_tracer.svg
- 01_injections_per_areas.svg
- 02_injection_site_volume_vs_tracers.svg
- 03_injection_site_volume_vs_tracers_log.svg
- 04_injection_site_volume_vs_extrinsic_cells_log.svg
- 03_injection_site_size_log.txt
- 04_injection_site_extrinsic_cells_log.txt

Figure S2: Relationship between the number of labeled neurons and injection site volume

```
cd S2_reproduce/
python 00_cells_vs_injection_size.py
```

Output:

- injections_size_vs_labelled_cells_total_cells_n.svg
- injections_size_vs_labelled_cells_extrinsic_cells_n.svg
- injections_size_vs_labelled_cells_intrinsic_cells_n.svg
- report_total_cells_n_CTBr.txt
- report_total_cells_n_CTBgr.txt
- report_total_cells_n_FR.txt
- report_total_cells_n_FE.txt
- report_total_cells_n_DY.txt
- report_total_cells_n_FB.txt
- report_extrinsic_cells_n_CTBr.txt
- report_extrinsic_cells_n_CTBgr.txt
- report_extrinsic_cells_n_FR.txt
- report_extrinsic_cells_n_FE.txt
- report_extrinsic_cells_n_DY.txt
- report_extrinsic_cells_n_FB.txt
- report_intrinsic_cells_n_CTBr.txt
- report_intrinsic_cells_n_CTBgr.txt
- report_intrinsic_cells_n_FR.txt
- report_intrinsic_cells_n_FE.txt
- report_intrinsic_cells_n_DY.txt
- report_intrinsic_cells_n_FB.txt

Figure S3: Calculation of geodesic paths and interareal distances

Panels C, E, F

```
cd S3_reproduce/
python 00_reproduce_wm_gm_comparison.py
python 01_histogram_interareal_distances.py
python 02_discrepancies.py
```

Output:

- distances_discrepancy.svg
- distribution_of_interareal_distances_20.00.svg
- speed_vs_distance_comparision.svg

Figure S4: Comparison of connectivity distance analysis using different definitions of primary areas

Panels A, B, C, D

```
cd S4_A-D_reproduce/
python 00_area_based_analysis.py
python 01_area_based_average_analysis.py.py
```

Output:

- gradient_areas_True_total_A3a_A3b_A4ab_A4c_AuA1_AuR_AuRT_V1.svg
- gradient_areas_True_total_A3a_A3b_AuA1_Gu_V1.svg
- gradient_areas_True_total_A3b_AuA1_AuR_V1.svg
- gradient_avg_areas_True_total_A3a_A3b_A4ab_A4c_AuA1_AuR_AuRT_V1.svg
- gradient_avg_areas_True_total_A3a_A3b_AuA1_Gu_V1.svg
- gradient_avg_areas_True_total_A3b_AuA1_AuR_V1.svg
- report_avg_True_total_A3b-A3a-AuA1-Gu-V1.txt
- report_avg_True_total_A3b-AuR-AuA1-V1.txt
- report_avg_True_total_AuRT-A4c-V1-A4ab-A3b-A3a-AuR-AuA1.txt
- report_True_total_A3b-A3a-AuA1-Gu-V1.txt
- report_True_total_A3b-AuR-AuA1-V1.txt
- report_True_total_AuRT-A4c-V1-A4ab-A3b-A3a-AuR-AuA1.txt

Panels E, F, G, H

```
cd S4_E-H_reproduce/
python 00_cell_based_analysis.py
python 01_cell_based_average_analysis.py
```

Output:

- gradient_avg_cell_True_total_A3a_A3b_A4ab_A4c_AuA1_AuR_AuRT_V1.svg
- gradient_avg_cell_True_total_A3a_A3b_AuA1_Gu_V1.svg
- gradient_avg_cell_True_total_A3b_AuA1_AuR_V1.svg
- gradient_cell_True_total_A3a_A3b_A4ab_A4c_AuA1_AuR_AuRT_V1.svg
- gradient_cell_True_total_A3a_A3b_AuA1_Gu_V1.svg
- gradient_cell_True_total_A3b_AuA1_AuR_V1.svg

- report__avg__True__total__A3b-A3a-AuA1-Gu-V1.txt
- report__avg__True__total__A3b-AuR-AuA1-V1.txt
- report__avg__True__total__AuRT-A4c-V1-A4ab-A3b-A3a-AuR-AuA1.txt
- report__True__total__A3b-A3a-AuA1-Gu-V1.txt
- report__True__total__A3b-AuR-AuA1-V1.txt
- report__True__total__AuRT-A4c-V1-A4ab-A3b-A3a-AuR-AuA1.txt

Licensing

This source code is distributed under the term of GNU General Public License v3 (GPL-3).
