# Supplementary figures and images for: Open access resource for cellular-resolution analyses of corticocortical connectivity in the marmoset monkey
Source: Nat Commun. 2020 Feb 28;11:1133. doi: 10.1038/s41467-020-14858-0 (PMC7048793; doi:10.1038/s41467-020-14858-0)

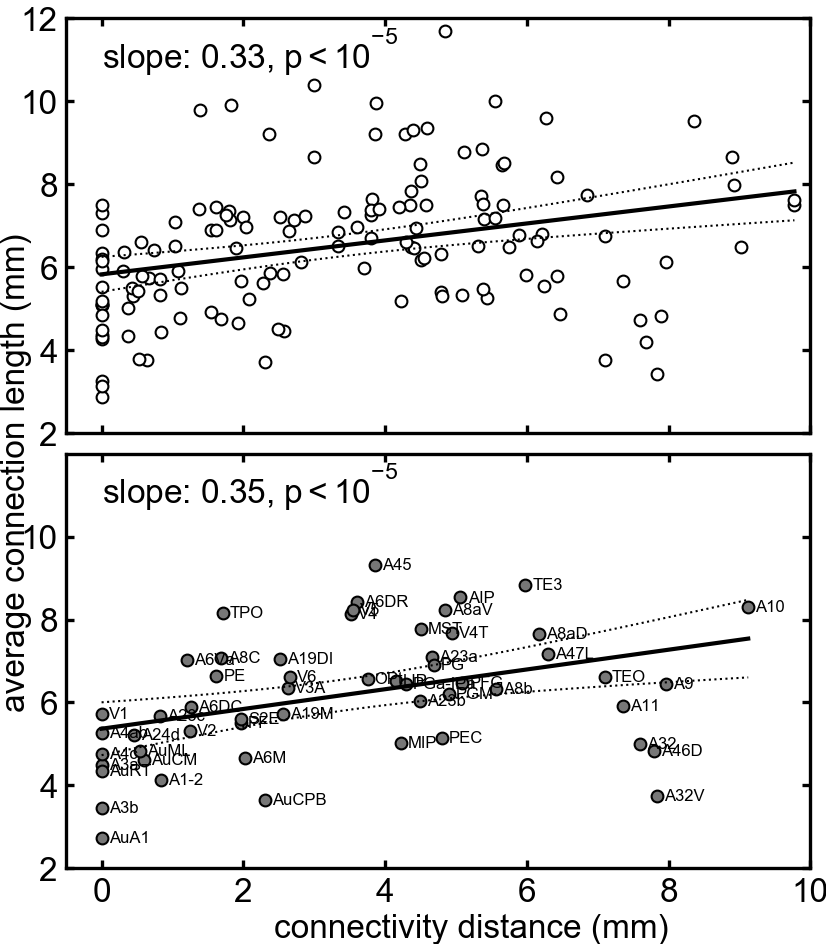

Supplement: Supplementary file 4 — Source Code [file 41467_2020_14858_MOESM4_ESM.zip › Source_Code/F5_AB_reproduce/gradient_areas_True_total_A3a_A3b_A4ab_A4c_AuA1_AuR_AuRT_V1.png]

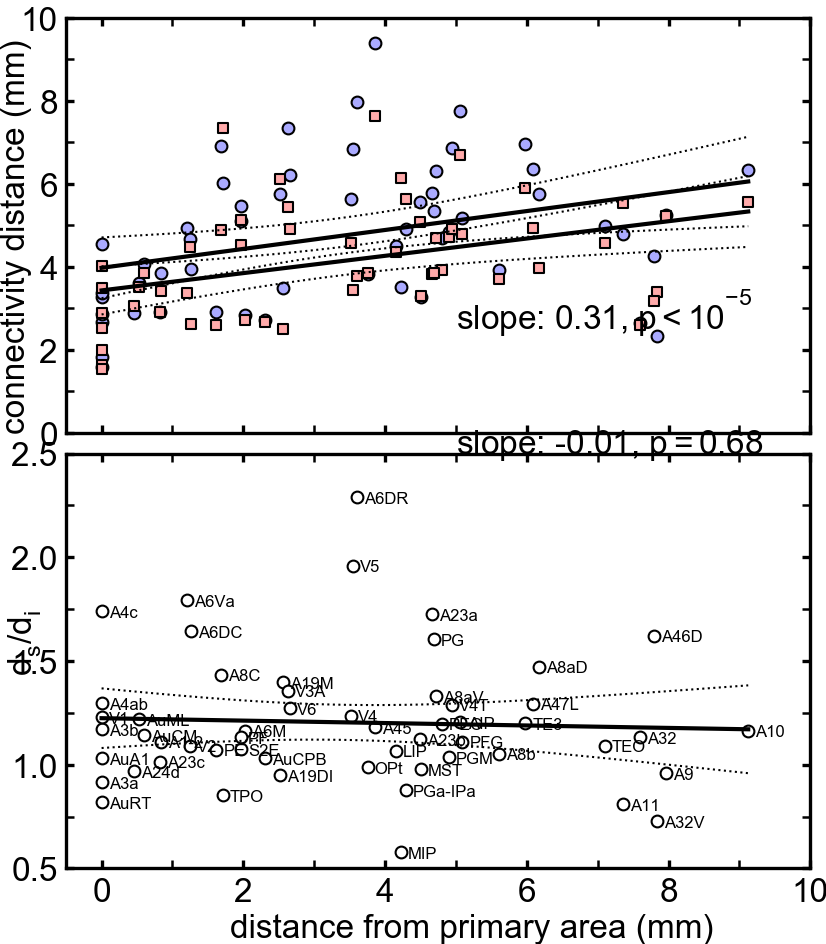

Supplement: Supplementary file 4 — Source Code [file 41467_2020_14858_MOESM4_ESM.zip › Source_Code/F5_CDEFG_reproduce/gradient_areas_True_ratio_A3a_A3b_A4ab_A4c_AuA1_AuR_AuRT_V1.png]

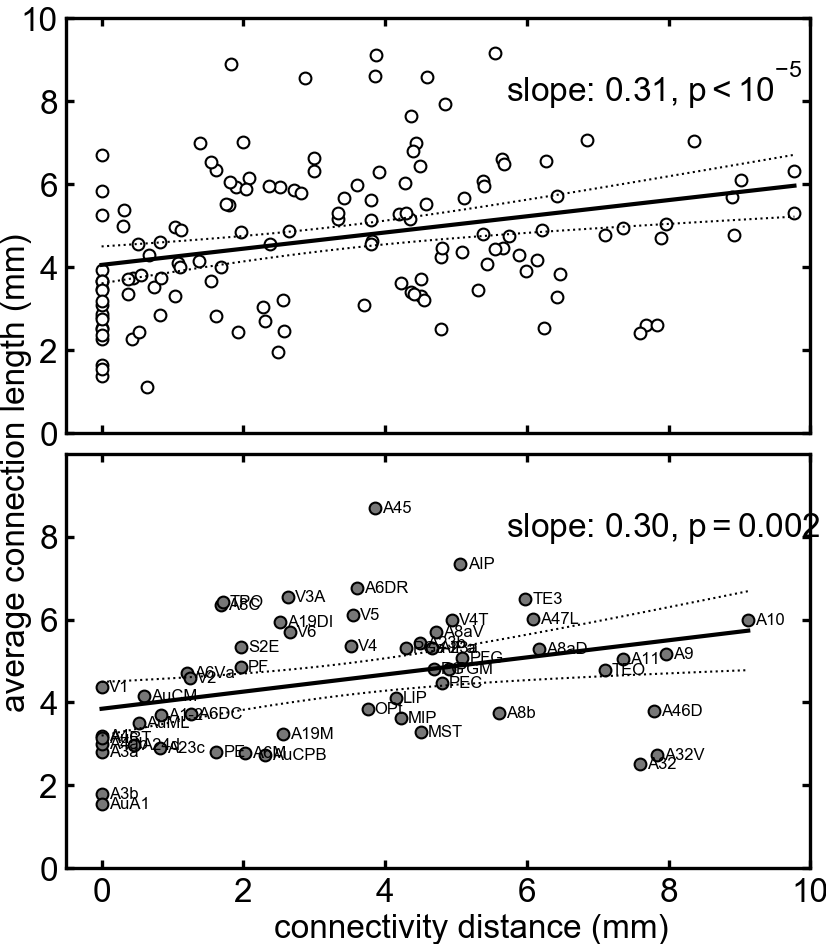

Supplement: Supplementary file 4 — Source Code [file 41467_2020_14858_MOESM4_ESM.zip › Source_Code/F5_CDEFG_reproduce/gradient_areas_True_total_A3a_A3b_A4ab_A4c_AuA1_AuR_AuRT_V1.png]

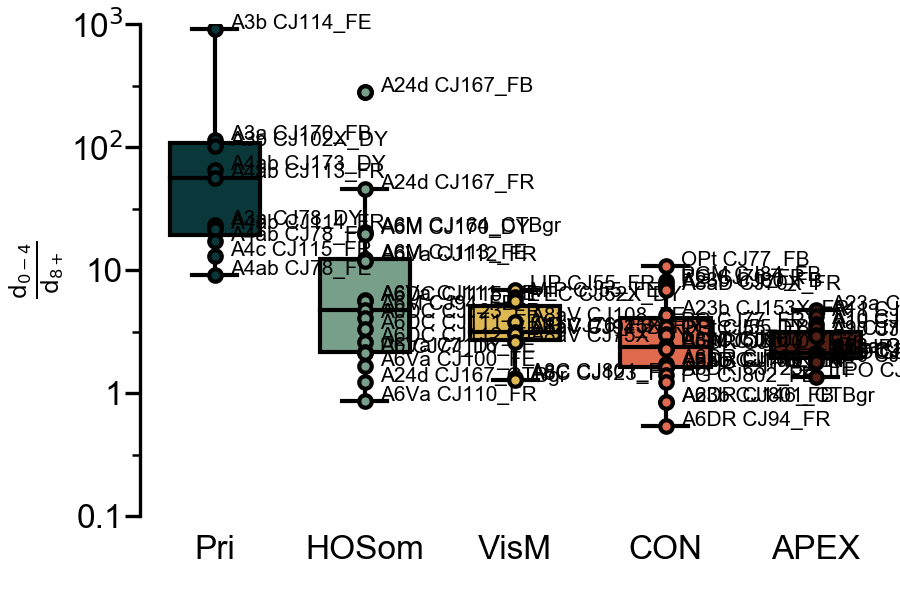

Supplement: Supplementary file 4 — Source Code [file 41467_2020_14858_MOESM4_ESM.zip › Source_Code/F7_reproduce/ratio.png]
